# The role of high fat diet on serum uric acid level among healthy male first degree relatives of type 2 diabetes mellitus

**DOI:** 10.1038/s41598-023-44843-8

**Published:** 2023-10-16

**Authors:** Dyah Purnamasari, Asri R. M. Umpuan, Christian Tricaesario, Wismandari Wisnu, Tri J. E. Tarigan, Dicky L. Tahapary, Muhadi Muhadi

**Affiliations:** 1 Division of Endocrinology Metabolism and Diabetes, Department of Internal Medicine, Cipto Mangunkusumo National Referral Hospital, Faculty of Medicine Universitas Indonesia, Jakarta, 10430 Indonesia; 2 Metabolic Disorder, Cardiovascular and Aging Research Center, The Indonesian Medical Education and Research Institute, Faculty of Medicine Universitas Indonesia, Jakarta, Indonesia; 3 Department of Internal Medicine, Cipto Mangunkusumo National Referral Hospital, Faculty of Medicine Universitas Indonesia, Jakarta, Indonesia; 4 Division of Cardiology, Department of Internal Medicine, Cipto Mangunkusumo National Referral Hospital, Faculty of Medicine Universitas Indonesia, Jakarta, Indonesia

**Keywords:** Metabolic syndrome, Type 2 diabetes, Risk factors

## Abstract

First-degree relatives (FDR) of type 2 diabetes mellitus have increased risk of developing insulin resistance-related disorders including hyperuricemia. We investigated metabolic profile and serum uric acid (SUA) metabolism in response to high-fat diet among healthy male FDR in comparison to those without family history of diabetes. A total of 30 FDR and 30 non-FDR subjects completed a 5-days-hypercaloric diet with fat added to regular daily intake. Despite similar insulin response, FDR displayed different changes in SUA compared to non-FDR subjects (0.26 ± 0.83 mg/dL vs − 0.21 ± 0.78 mg/dL, p = 0.028). In subgroup analyses stratified by body mass index and waist circumference, significant different SUA changes between FDR and non-FDR subjects were only found in obese (0.48 ± 0.87 mg/dL vs − 0.70 ± 0.71 mg/dL, p = 0.001) and centrally obese (0.59 ± 0.83 mg/dL vs − 0.55 ± 0.82 mg/dL, p = 0.011) subgroups. In multivariate analysis, visceral adiposity seemed mediating the different response in SUA metabolism between FDR and non-FDR subjects induced by short-term obesogenic diet.

## Introduction

Type 2 diabetes mellitus (T2DM) is a global major health problem leading to morbidity, premature mortality, and economic burden in healthcare system^1^. One of risk factors of T2DM is being a first-degree relative (FDR) of T2DM, which increases the odds of conversion to T2DM by two- to sevenfold compared to those without parental history of T2DM^2^. The tendencies of FDR to have higher body mass index (BMI), lower insulin sensitivity and defective early-phase insulin release representing ß-cell are proposed to explain this susceptibility^2,3^. It also has been observed that FDR had increased risk of having insulin resistance-related disorders such as metabolic syndrome which includes glycemic dysregulation, dyslipidemia, central obesity, hypertension, and also hyperuricemia^2–5^.

Hyperuricemia is an abnormality of uric acid metabolism which results in a condition of excessive serum uric acid (SUA) as a result of purine metabolism^6,7^. Previous studies showed that hyperuricemia takes part to the process of insulin resistance bidirectionally, being both as causal and end-product of insulin resistance^8–10^. Both insulin resistance and SUA were associated with the burden of inflammatory conditions including T2DM^10^, hypertension^11^, non-alcoholic fatty liver disease^12^. Furthermore, SUA and its variability have been reported to be associated with diabetes complications including cardiovascular events, cerebral infarction, cardiovascular mortality, and diabetic kidney disease on people with T2DM^13–17^. Despite the numerous studies regarding SUA in T2DM, little is known in FDR population. Few studies observed conflicting results regarding SUA level in FDR compared to those without family history of T2DM^5,18,19^. Even so, FDR population with hyperuricemia was found to already have endothelial dysfunction even in normoglycemic state^20^. Therefore, SUA has been proposed as a biomarker to predict insulin resistance and T2DM.

Obesogenic milieu favoring inflammatory state such as high-fat diet is associated with weight gain and its adverse metabolic effects, such as increase in body weight and insulin resistance, which are more pronounced in FDR population^21,22^. Higher dietary fat intake has also been associated with higher level of SUA in a large general population-based cross-sectional study^23^. While the effects of various dietary carbohydrate and protein modifications on SUA and other metabolic parameters in T2DM have been much studied^24^, the effect of hypercaloric diet with high content of fat on SUA was rarely reported. Taken together, we aimed to see how this obesogenic diet in form of short-term high-fat diet alters metabolic profiles including SUA and insulin resistance in healthy young adult male FDR population in comparison to healthy young adult male counterpart without parental history of T2DM.

## Methods

### Study design and participants

This is a non-randomised, prospective comparative study including healthy male aged 25–39 years, assigned as FDR of T2DM subjects and non-FDR of T2DM as control group. The FDR of T2DM was recruited consecutively from the offspring of T2DM patients treated in Endocrinology and Diabetes outpatient clinic at Cipto Mangunkusumo National Referral Hospital in Jakarta, Indonesia. While non-FDR of T2DM was recruited consecutively from medical and non-medical staffs who did not have history of T2DM in the first- and second-degree levels. Subjects with history of smoking, taking diuretic medications, oral contraception or other medications affecting uric acid, lipid or glucose metabolism, body mass index (BMI) ≥ 35 kg/m^2^, impaired glucose tolerance, and hypertension were excluded from the study. The Ethics Committee of Faculty of Medicine Universitas Indonesia/Cipto Mangunkusumo National Referral Hospital has evaluated and approved the study protocol (KET-1100/UN2.F1/ETIK/PPM.00.02/2020). This study was performed in accordance with the principles of Declaration of Helsinki and all methods were carried out in accordance with relevant guidelines and regulations. All subjects gave written informed consent before enrollment.

### Clinical evaluation and intervention

Subjects who suited inclusion criteria then underwent physical examinations and blood collections for measurement of fasting blood glucose (FBG) and HbA1C. Blood pressure was measured with automatic blood pressure monitor (HEM-7121, Omron Healthcare Co, Ltd, Kyoto, Japan). Fasting blood glucose for eligibility screening and HbA1C were measured with point of care testing using AccuChek Performa (Roche Diabetes Care, Inc., Indianapolis, IN, US) and A1C Glycohemoglobin Analyzer EZ test 2.0 (BioHermes Biomedical Technology Co., Ltd., Wuxi, China), respectively (normoglycemic if FBG < 100 mg/dL and HbA1C < 5.7%). Subjects with BMI 18.5–34.9 kg/m^2^, normal glucose tolerance and normal blood pressure proceeded to intervention phase on different day.

Subjects were examined on the day before high-fat diet (HFD) intervention (D-0) by having their blood pressure and waist circumference measured and blood samples taken after an overnight fasting (10–12 h of fasting). Five packs of 250 mL liquid whipping cream (Anchor whipping cream, Fonterra™ Brands Indonesia Ltd, Jakarta, Indonesia) were given, which would be taken one pack a day [837.5 kcal/day, containing 95% fat (60% saturated fat)] as HFD supplementing normal daily intake for both groups for 5 consecutive days. Subjects were instructed to keep their regular daily intake and write food diary in addition to daily video call checking to maintain adherence. One day after the fifth day of HFD (D-6), subjects were asked to come bringing the food diary and to have their blood drawn after an overnight fasting (10–12 h). Metabolic parameters measured from blood taken before and after HFD were fasting blood glucose, fasting insulin, and SUA. Dietary assessment using 24 h food-recall was performed before and during HFD intervention by registered and trained dieticians for 3 days, consisted of 2 working days and 1 weekend day or holiday.

### Laboratory measurements

Serum uric acid and fasting blood glucose before and after HFD intervention were tested with laboratory procedure using RocheCobas 501 MPA® with enzymatic colorimetry method. Fasting insulin level was measured by chemiluminescent microparticle immunoassay (CMIA). Insulin resistance was presented as HOMA-IR and calculated with formula: FBG [mg/dL] × fasting insulin [μIU/mL]/405^25^.

### Statistical analysis

Analysis was done using IBM SPSS version 25. Shapiro–Wilk test was used to analyze data distribution. Normally distributed data were presented as mean ± standard deviation, while non-normally distributed data were presented as median (interquartile range). For normally distributed data, the independent t-test was used to analyze mean difference and paired t-test was used to analyze pre- and post-intervention difference. Mann–Whitney test and Wilcoxon signed-rank test were used for non-normally distributed data. Baseline characteristics were further stratified by age cut-off of 30 years, while pre- and post- intervention differences were further analyzed based on BMI and waist circumference. Multivariate analysis was conducted using linear regression to adjust FDR status association with SUA changes for age, fat intake alteration, carbohydrate intake alteration, protein intake alteration, sucrose intake alteration, HOMA-IR changes, BMI, and waist circumference.

## Results

### Baseline characteristics

There were 65 male subjects recruited to our study, consisted of 32 FDR subjects and 33 non-FDR subjects. We observed higher waist circumference (87.5 ± 11.1 cm vs 80.8 ± 10.5 cm, p = 0.014) in FDR compared to non-FDR subjects, but no significant differences were found in BMI (p = 0.168), fasting insulin (p = 0.454), HOMA-IR (p = 0.304), HbA1C (p = 0.526), and FBG (p = 0.083) in baseline characteristics (Table 1). There was no difference of baseline SUA levels between both groups (6.7 ± 1.1 mg/dL and 6.6 ± 1.6 mg/dL, p = 0.757). Stratified by age, the differences between FDR in comparison with non-FDR in waist circumference was remained significant in subgroup of 30 years or older whereas in those aged < 30 years did not have significant difference in all baseline parameters (Table S1). In FDR group aging ≥ 30 years, systolic blood pressure (128 ± 7 mmHg vs 118 ± 8 mmHg) and SUA level (7.4 ± 1.3 mg/dL vs 6.5 ± 0.9 mg/dL) were significantly higher than those in FDR aging < 30 years with p < 0.01 and p < 0.05, respectively, whereas in non-FDR population there were no significant differences (Table S1).

**Table 1:** Baseline characteristics of study participants.

Variables	All subjects (N = 65)	FDR (N = 32)	Non-FDR (N = 33)	P
Age (years)	28 (26–31)	28 (26–31)	28 (26–34)	0.968
Body mass index (kg/m^2^)	24.2 (22.1–26.9)	24.9 (22.6–27.9)	23.3 (21.5–25.5)	0.168
Waist circumference (cm)	84.1 ± 11.2	87.5 ± 11.1	80.8 ± 10.5	0.014*
Systolic pressure (mmHg)	122 ± 8	121 ± 9	123 ± 7	0.367
Diastolic pressure (mmHg)	81 ± 7	81 ± 8	81 ± 7	0.689
Fasting blood glucose (mg/dL)	82 (77–88)	85 (79–88)	81 (76–87)	0.176
Fasting insulin (μIU/mL)	7.5 (5.7–9.9)	7.9 (6.3–10.4)	7.2 (5.3–9.7)	0.454
HOMA-IR	1.66 ± 0.71	1.75 ± 0.78	1.57 ± 0.63	0.304
HbA1c (%)	5.1 ± 0.3	5.2 ± 0.3	5.1 ± 0.3	0.526
HbA1c (mmol/mol)	33 ± 3	33 ± 4	32 ± 3	0.526
Serum uric acid (mg/dL)	6.7 ± 1.3	6.7 ± 1.1	6.6 ± 1.6	0.757

All values are expressed in mean ± SD or median (IQR).

*FDR* first-degree relatives of type 2 diabetes mellitus, *HOMA-IR* homeostatic model assessment for insulin resistance, *HbA1C* glycated hemoglobin.

*p < 0.05.

### High-fat diet intervention dietary assessment

**Figure 1:**
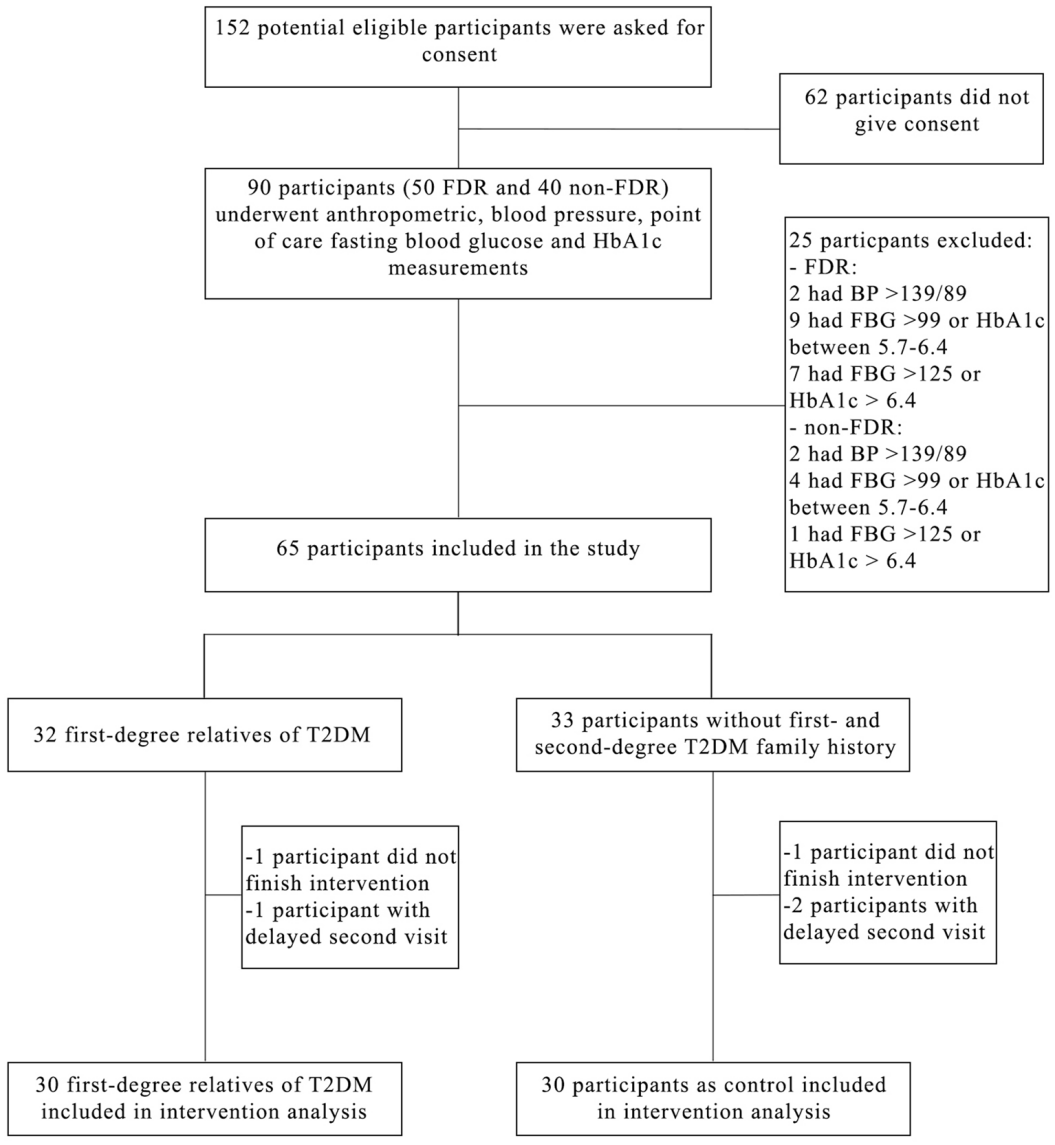
Participant recruitment flow. *BP* blood pressure, *FBG* fasting blood glucose, *FDR* first-degree relatives of type 2 diabetes mellitus, *T2DM* type 2 diabetes mellitus.

After starting HFD intervention, 2 subjects (1 FDR and 1 non-FDR) did not finish the intervention and 3 subjects (1 FDR and 2 non-FDR) postponed their post-HFD examination to D-7, thereby we excluded these 5 subjects from intervention analysis (Fig. 1). Dietary intake analysis before HFD intervention revealed that both groups had similar average energy intake, where fat intake contributed around 36.6% and 34.4% of total daily energy intake in FDR and non-FDR groups, respectively (Table S2). During HFD intervention, energy gained from fat increased in both groups to around 54.4% and 57.2% in FDR and non-FDR groups, respectively indicating the intervention was sufficient to achieve HFD. Sucrose intake were also similar between both group at baseline and during intervention (Table S2). Good compliance to HFD intervention was observed, as also showed by increase in total energy intake to almost 60% compared to baseline intake. There was also no decrease in carbohydrate and protein intake in both groups during HFD intervention compared to baseline (Table S2).

### Metabolic responses after HFD

**Figure 2:**
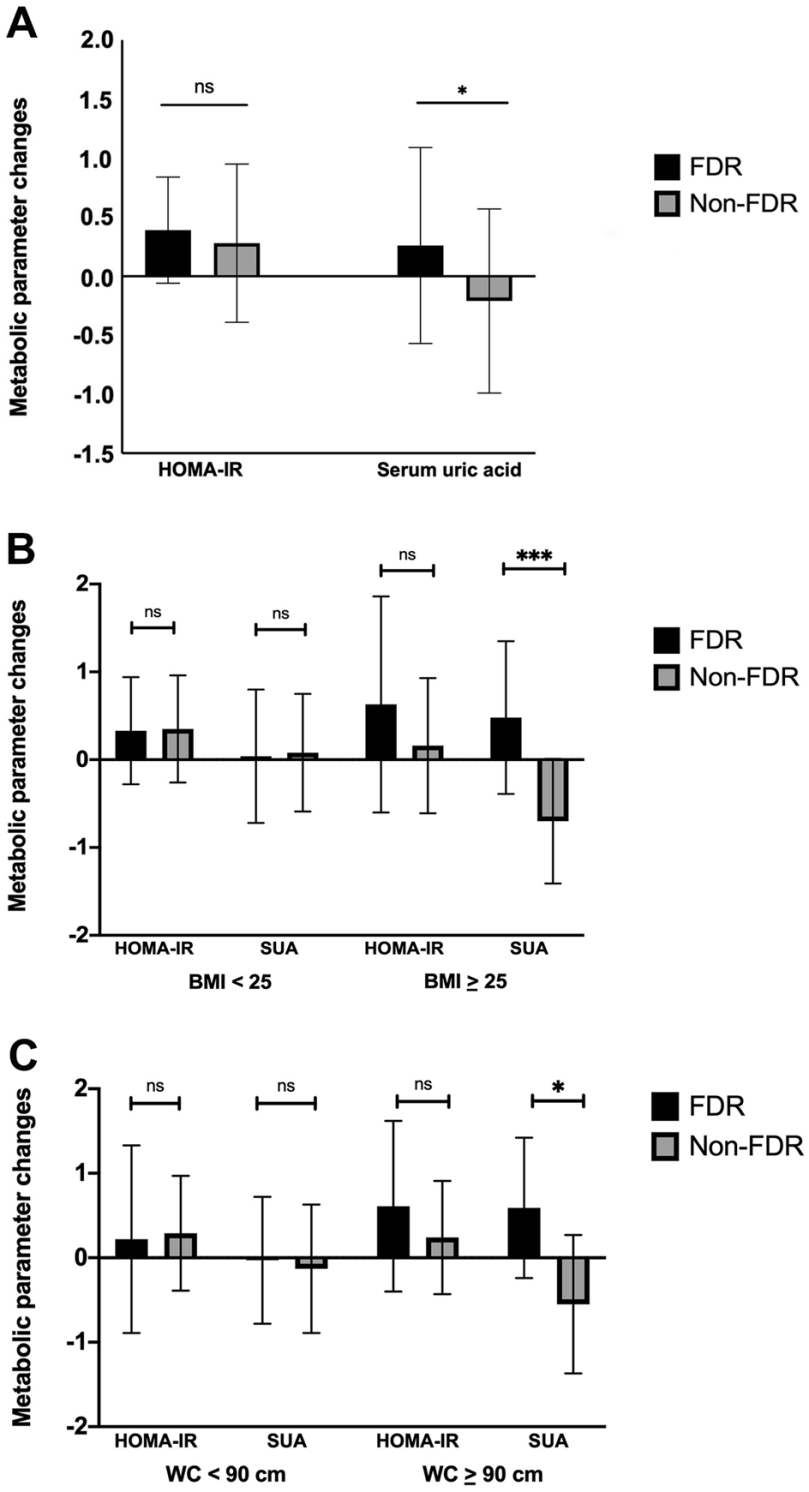
The comparison of HOMA-IR and serum uric acid changes as response to high-fat diet: overall comparison (**A**), comparison stratified by BMI cut-off of 25 kg/m^2^ (**B**), and stratified by waist circumference of 90 cm (**C**). *BMI* body mass index, *FDR* first-degree relatives of type 2 diabetes mellitus, *HOMA-IR* homeostatic model assessment for insulin resistance, *SUA* serum uric acid, *WC* waist circumference (*p < 0.05; **p < 0.01; ***p < 0.001; ns: p > 0.05).

Responding to 5 days-HFD interventions, both FDR and non-FDR groups showed increases in insulin levels (7.85 [6.35–10.13] μIU/mL to 9.25 [6.78–13.90] μIU/mL, p = 0.008 and 7.30 [5.30–9.53] to 8.00 [6.88–10.73] μIU/mL, respectively, p = 0.009) and HOMA-IR (1.64 [1.18–2.12] to 1.85 [1.43–3.03], p = 0.010 and 1.52 [1.19–1.88] μIU/mL to 1.64 [1.33–2.21] μIU/mL, respectively, p = 0.027) (Table 2). Nonetheless, the difference between two groups was not significant. In terms of weight gain, FDR group showed similar weight gain percentage compared to those of non-FDR (0.6 ± 1.0% vs 0.7 ± 1.3%, p = 0.771). On the other hand, despite the non-significant changes in SUA in both groups, the response was significantly different (p = 0.028) when both groups were compared, as FDR subjects had mean difference of 0.26 ± 0.83 mg/dL whereas non-FDR subjects had mean difference of − 0.21 ± 0.78 mg/dL (Fig. 2A).

**Table 2:** Metabolic profile before and after HFD intervention.

	FDR (N = 30)				Non-FDR (N = 30)				P between groups	
	Pre-HFD	Post-HFD	P^a^	Magnitude of changes	Pre-HFD	Post-HFD	P^a^	Magnitude of changes	Post-HFD^b^	Magnitude of changes^c^
Body weight (kg)	71.8 ± 12.6	72.2 ± 12.6	0.004**	0.4 ± 0.8	66.1 ± 10.0	66.6 ± 10.0	0.007**	0.4 ± 0.8	0.061	0.948
FBG (mg/dL)	84 ± 6	86 ± 9	0.369	1.5 ± 8.9	83 ± 9	82 ± 9	0.776	− 0.4 ± 7.0	0.139	0.374
Fasting insulin (μIU/mL)	7.85 (6.35–10.13)	9.25 (6.78–13.90)	0.008**	1.40 (− 0.13 to 4.00)	7.30 (5.30–9.53)	8.00 (6.88–10.73)	0.009**	1.05 (− 0.10 to 3.23)	0.408	0.599
HOMA-IR	1.64 (1.18–2.12)	1.85 (1.43–3.03)	0.010*	0.39 (− 0.08 to 0.71)	1.52 (1.19–1.88)	1.64 (1.33–2.21)	0.027*	0.16 (− 0.07 to 0.60)	0.344	0.530
SUA (mg/dL)	6.8 ± 1.1	7.0 ± 1.4	0.098	0.26 ± 0.83	6.7 ± 1.6	6.5 ± 1.3	0.155	− 0.21 ± 0.78	0.102	0.028*

All values are expressed in mean ± SD or median (IQR).

*FDR* first-degree relatives of type 2 diabetes mellitus, *HFD* high-fat diet, *FBG* fasting blood glucose, *HOMA-IR* homeostatic model assessment for insulin resistance, *SUA* serum uric acid.

*p < 0.05, **p < 0.01.

^a^Paired t-test or Wilcoxon signed-rank test of mean difference before and after high-fat diet intervention.

^b^Independent t-test or Mann–Whitney test of mean difference after high-fat diet intervention between FDR vs non-FDR.

^c^Independent t-test or Mann–Whitney test of metabolic parameters changes (after HFD–before HFD) difference between FDR vs non-FDR.

Stratified by BMI, subjects with BMI ≥ 25 exhibited a significantly different changes in SUA when FDR was compared to non-FDR counterpart (0.48 ± 0.87 mg/dL vs − 0.70 ± 0.71 mg/dL, p = 0.001) (Fig. 2B, Table S3). Dividing subjects based on waist circumference, SUA changes was also significantly different between FDR and non-FDR subjects in those with central obesity (0.59 ± 0.83 mg/dL vs − 0.55 ± 0.82 mg/dL, p = 0.011) (Fig. 2C, Table S4).

In multivariate analysis, adjusting for age, dietary (fat, carbohydrate, protein, sucrose) alterations, HOMA-IR changes, and BMI, FDR status was consistently remained its significant positive association with SUA changes (β 0.480, 95% CI 0.014–0.947, p = 0.044) (Table 3). However, when waist circumference was added as covariate, its association became not significant (β 0.442, 95% CI − 0.039 to 0.924, p = 0.071) (Table 3).

**Table 3:** Multivariate analysis of serum uric acid changes association with FDR status in response to HFD.

Independent variable	Model	β (95% CI)	*P* value
FDR status	Model 1	0.465 (0.045 to 0.884)	0.030*
	Model 2	0.487 (0.031 to 0.942)	0.037*
	Model 3	0.506 (0.044 to 0.968)	0.032*
	Model 4	0.480 (0.014 to 0.947)	0.044*
	Model 5	0.442 (− 0.039 to 0.924)	0.071

Linear regression with FDR status as independent variable where β value denotes serum uric acid change difference of the FDR group had in comparison to non-FDR group.

Model 1: Adjusted for age.

Model 2: Adjusted for age, fat intake alteration, carbohydrate intake alteration, protein intake alteration, and sucrose intake alteration.

Model 3: Adjusted for age, fat intake alteration, carbohydrate intake alteration, protein intake alteration, sucrose intake alteration, and HOMA-IR changes.

Model 4: Adjusted for age, fat intake alteration, carbohydrate intake alteration, protein intake alteration, sucrose intake alteration, HOMA-IR changes, and body mass index.

Model 5: Adjusted for age, fat intake alteration, carbohydrate intake alteration, protein intake alteration, sucrose intake alteration, HOMA-IR changes, and waist circumference.

*FDR* first degree relatives of type 2 diabetes, *HFD* high-fat diet.

*Values denote statistical significance at* p* < 0.05.

## Discussion

This study involved normoglycemic, normotensive young adult FDR of T2DM population without history of smoking. Different to previous similar studies which include subjects with hypertension, impaired glucose tolerance, and smoking history, this study would provide an interesting point of detecting metabolic alteration in the earlier stage on high-risk population of developing T2DM^2,4,26^. By analyzing the SUA profile from healthy subjects as such, we aimed that the results would highlight the association of parental history and metabolic dysregulations.

Our study showed that compared to general population, male FDR population had higher waist circumference which was more pronounced in those aged older than 30 years. At baseline, insulin resistance as expressed by HOMA-IR and SUA were similar between FDR and non-FDR subjects. Intervention given was sufficient to achieve HFD which is defined as diet consisting of at least 35% of total calories is consumed from fats^27^, where in previous studies typically contain 32–60% of calories from fat^28^. Responding to HFD intervention, despite similar increase in HOMA-IR displayed by both groups, significant different changes in SUA was observed between FDR and non-FDR subjects, as SUA slightly increased in FDR subjects, whereas it slightly decreased in non-FDR subjects. This different manner was more clearly observed in obese and centrally obese subjects. In general, the change in SUA might be mediated by abdominal adiposity, while in obese subjects, FDR status independently associated with SUA change in response to HFD.

It has been known that FDR population tend to have higher adiposity presented by higher BMI and waist circumference compared to general population^3,4,29^. Interestingly, in current study we found that FDR had higher waist circumference within the similar BMI range in comparison to non-FDR subjects. This difference was mainly contributed by subjects aged 30 years or more. Being FDR was reported to have inappropriately hypertrophic subcutaneous adipose tissue despite the normal BMI, which leads to ectopic fat accumulation such as in intraabdominal fat depot (which clinically measured by waist circumference) due to adipose tissue dysregulation^30^. As men age, reduced physical activity, lower basal metabolic rate, and decline in testosterone contribute to redistribution of fat to visceral adipose tissue^31^. This study observed that in subjects aging 25–30 years, those having FDR of T2DM still had similar waist circumference to those of non-FDR counterpart. Meanwhile FDR subjects aging 30–39 years had significantly higher abdominal adiposity compared to corresponding non-FDR, suggesting susceptibility to earlier adipose tissue disturbance than general population within the same phase of aging, though it should be noted that we included small sample size especially after stratifying based on age.

In this study, FDR had similar SUA level to those of non-FDR group at baseline and their mean value was in normal range. There were only very few studies comparing SUA level between FDR and non-FDR subjects, all of which gave conflicting results. Mohan et al.^5^ and van der Sande et al.^19^ found higher SUA level in FDR population compared to non-FDR population, whereas González-Ortíz et al.^18^ did not observe difference in SUA level between both group. While González-Ortíz recruited young subjects aging 19–20 years that more resembled our subject characteristics, both Mohan and van der Sande included subjects of older age compared to our study which might contribute to the contradicting result. Moreover, van der Sande included inequally very large number of non-FDR subjects in regards to the FDR subject, which might also affect the result in addition to overpowered sample size. Indeed, age has been associated with increased SUA and thereby might explain the difference found between our study and previous reports^32,33^. Interestingly, we found that in FDR subjects who were on their fourth decade of life had significantly higher SUA than those on the third decade, while it was not observed in non-FDR subjects. This implicated that genetic susceptibilty relating to FDR might play a role to some extent beside the influence of aging and abdominal obesity^9^.

As expected, HFD induced increase in insulin secretion and thus increased insulin resistance as presented by HOMA-IR, though the increment was similar among both groups. It was in line with previous studies which also observed increase in insulin resistance in response towards HFD interventions^21,22,34^. Underlying mechanisms by which HFD results in insulin resistance remains not fully understood. It is suggested that short-term HFD induced hepatic and muscle insulin resistance, possibly as consequences of hepatic fat accumulation through diacylglyecrol and ceramide-induced insulin signaling inhibition, elevated gastric inhibitory polypeptide secretion, and increased gene expression of pro-inflammatory macrophages leading to the release of pro-inflammatory cytokines that impairs hepatic insulin signaling^22,35,36^. There was one study investigating 28 days of HFD impact in FDR population compared to non-FDR which found that the increase of HOMA-IR in FDR was significantly higher than those of non-FDR subjects, even though there was not found any difference in any body fat parameter changes between both groups^21^. The difference between previous study and our study might be contributed by the strict diet menu provided by the investigator in previous study which controlled subjects daily dietary intake from 3 days prior to HFD intervention to the end of day 28 of intervention. They also gave higher additional energy intake to the subjects and included older subjects with higher BMI compared to our study.

We observed similar post-HFD SUA level compared to baseline in both groups. Previous studies have demonstrated association between fat intake and increase in SUA level in general population and animal model^23,37,38^. In contrast, Oku et al.^39^ found that dietary fat intake had no impact on SUA level in normal population, but associated with lower risk of high SUA level in men with chronic kidney disease. High-fat diet is thought to affect SUA by increasing vascular xanthine oxidase activity and decreasing the excretion of uric acid into urine due to acute hyperinsulinemia or insulin resistance and due to increase of ketone bodies^37,40,41^.

In spite of the similar increase in insulin resistance between both groups, FDR subjects showed different trend of SUA changes compared to non-FDR subjects in response to HFD. It has been reported that adipose tissue especially of visceral origin has xanthine oxidoreductase activities which produce uric acid and was pronounced in obese subjects including in HFD-induced obesity^42,43^. Indeed compared with non-FDR subjects, FDR subjects had higher waist circumference as clinical marker of visceral adiposity which might explain the difference in SUA metabolism response towards HFD. Stratified by BMI and waist circumference, as expected, the difference in SUA metabolism response was influenced by higher adiposity, as obese and centrally obese FDR showed significant difference in SUA metabolism response in comparison to non-FDR counterpart, whereas the normoweight subgroups did not. Multivariate analyses further confirmed this association, in which the association between being an FDR with SUA change in response to HFD became not significant when waist circumference was added to linear regression model after adjusting to age, dietary intake alterations, HOMA-IR changes and BMI. It implies that beside the previous evidences reporting baseline SUA level is determined by visceral adiposity, we found that HFD induced different SUA level change in FDR compared to non-FDR that was mediated by visceral adiposity, but not BMI. However, our linear regression model did not fully explain the SUA changes, suggesting other factors other than visceral adiposity also played a role, one of which might be genetic traits. It has been observed that SUA is heritable and that polymorphism in ABCG2 and SLC2A9 genes played important roles to SUA regulation^44,45^. Therefore, further studies investigating the relation between uric acid-related polymorphism and FDR are needed to elucidate the underlying mechanism.

This research is the first to study the role of HFD in SUA level in young adult healthy male of high-risk population towards T2DM, notably FDR of T2DM with non-FDR subjects as control group. Thus, this study might provide insights regarding preclinical metabolic disorders, particularly in response to obesogenic diet, in Indonesian FDR population who were clinically healthy as early as possible. However, there are limitations in our study. First, this was a single center study which included small sample size, particularly for the cross-sectional analysis at baseline. Furthermore, only male subjects were recruited thereby the results might not be applicable for female FDR population as there are hormonal factors which play role in premenopausal women. Next, we did not have purine intake data which is also an important factor affecting SUA level. We also only performed HFD intervention for 5 days, so the effect of HFD in long-term needs further studies. Finally, we did not strictly controlling subjects daily intake that might result in slight difference between groups despite the achieved HFD condition, yet this might be also the strength of our study as it can represent real-world individual variation of food intake.

In conclusion, baseline SUA level in normoglycemic and normotensive young adult male FDR of T2DM was in normal range and was not different from those in the non-FDR group. Short-term HFD intervention induced similar increase in insulin resistance among both groups, but different changes in SUA level regardless the normal and similar baseline SUA level, which might be mediated by visceral adiposity, hence the more prominent difference in obese and centrally obese male subjects. However, larger studies including female subjects with longer duration of HFD intervention and genetic analysis are needed to see the role of chronic obesogenic diet to SUA level and the genetic traits involved as underlying mechanism in FDR population, and how these changes affect their risk for further metabolic disorders.

### Supplementary Information

Supplementary Tables.

## Data Availability

All data generated or analysed during this study are included in this published article (and its Supplementary Information files).
