# Supplementary material for: The role of high fat diet on serum uric acid level among healthy male first degree relatives of type 2 diabetes mellitus
Source: Sci Rep. 2023 Oct 16;13:17586. doi: 10.1038/s41598-023-44843-8 (PMC10579419; doi:10.1038/s41598-023-44843-8)
Supplement: Supplementary file 1 — Supplementary Tables. [file 41598_2023_44843_MOESM1_ESM.pdf]

SUPPLEMENTARY TABLES

The role of high fat diet on serum uric acid level among healthy male first degree relatives of type 2 diabetes mellitus

Dyah Purnamasari[1,2,*], Asri R. M. Umpuan[3], Christian Tricaesario[2], Wismandari Wisnu[1,2], Tri J. E. Tarigan[1,2], Dicky Levenus Tahapary[1,2], Muhadi Muhadi[4]

[1] Division of Endocrinology Metabolism and Diabetes, Department of Internal Medicine, Cipto Mangunkusumo National Referral Hospital, Faculty of Medicine Universitas Indonesia, Jakarta, Indonesia

[2] Metabolic Disorder, Cardiovascular and Aging Research Center, The Indonesian Medical Education and Research Institute, Faculty of Medicine Universitas Indonesia, Jakarta, Indonesia

[3] Department of Internal Medicine Department, Cipto Mangunkusumo National Referral Hospital, Faculty of Medicine Universitas Indonesia, Jakarta, Indonesia

[4] Division of Cardiology, Department of Internal Medicine, Cipto Mangunkusumo National Referral Hospital, Faculty of Medicine Universitas Indonesia, Jakarta, Indonesia

Supplementary table S1. Baseline characteristics stratified by age categories

Variables	Age < 30			Age ≥ 30		
	FDR (N=23)	Non-FDR (N=20)	P	FDR (N=9)	Non-FDR (N=13)	P
Body mass index (kg/m^2)	24.3 (19.6-28.2)	23.1 (21.3-25.0)	0.318	25.4 (24.3-27.2)	24.6 (22.0-27.1)	0.510
Waist circumference (cm)	85.99±12.08	81.35±10.15	0.184	91.52±7.06	79.95±11.35	0.014[a]
Systolic pressure (mmHg)	116 (112-126)	122 (118-129)	0.105	129 (121-135)[b]	128 (121-131)	0.292
Diastolic pressure (mmHg)	80.0±8.1	80.2±6.6	0.916	84.7±6.0	81.1±7.6	0.252
Fasting blood glucose (mg/dL)	84.1±7.2	81.9±7.2	0.325	83.0±5.7	82.3±10.3	0.857
Fasting Insulin (μIU/mL)	8.80±4.38	8.12±3.11	0.561	7.68±2.06	7.15±3.14	0.667
HOMA-IR	1.82±0.88	1.63±0.57	0.413	1.58±0.43	1.48±0.74	0.715
HbA1C (%)	5.09±0.35	5.08±0.31	0.906	5.31±0.24	5.14±0.30	0.161
Serum uric acid (mg/dL)	6.45±0.87	6.57±1.26	0.710	7.39±1.34[b]	6.67±1.98	0.354

All values are expressed in mean±SD or median (IQR)

[a]Independent t-test or Mann-Whitney test for mean difference between FDR vs non-FDR aging ≥ 30 years with values denoting $p<0.05$

[b]Independent t-test or Mann-Whitney test for mean difference between FDR aging $\geq$ 30 years vs FDR aging < 30 years with values denoting $p<0.05$

FDR, first-degree relatives of type 2 diabetes mellitus; HOMA-IR, homeostatic model assessment for insulin resistance; HbA1C, glycated hemoglobin

Supplementary table S2. Dietary intake analysis before and during high-fat diet intervention

Variables	FDR (N=30)			Non-FDR (N=30)			P[b]	P[c]	P[d]
	Before HFD	During HFD	P[a]	Before HFD	During HFD	P[a]			
Total energy intake (kcal/day)	1,581±293	2,490±368	<0.001***	1,490±414	2,375±366	<0.001***	0.332	0.231	0.844
Energy from carbohydrate (kcal/day)	736 (680-883)	910 (651-1,053)	0.008**	744 (645-839)	807 (689-956)	0.329	0.918	0.298	0.323
Energy from protein (kcal/day)	238 (203-266)	264 (210-303)	0.081	206 (140-258)	228 (188-262)	0.165	0.132	0.040*	0.797
Energy from fat (kcal/day)	549 (482-717)	1,377 (1,270-1,454)	<0.001***	525 (409-637)	1,341 (1,219-1,474)	<0.001***	0.110	0.501	0.367
Percentage of energy from fat (%)	36.6 (33.4-40.5)	54.4 (53.0-60.5)	<0.001***	34.7 (30.6-38.2)	57.9 (52.2-61.4)	<0.001***	0.044*	0.352	0.005**
Sucrose (g)	14.3 (7.1-26.3)	16.4 (8.4-30.8)	0.420	18.4 (10.6-25.3)	16.3 (12.6-22.2)	0.894	0.322	0.701	0.712

All values are expressed in mean±SD or median (IQR)

[a]Paired t-test or Wilcoxon signed-rank test of before and during high-fat diet intervention mean difference

[b]Independent t-test or Mann-Whitney test for mean difference of baseline dietary intake between FDR vs non-FDR

[c] Independent t-test or Mann-Whitney test for mean difference of dietary intake during HFD between FDR vs non-FDR

[d]Independent t-test or Mann-Whitney test for mean difference of dietary intake changes (during - before high-fat diet) between FDR vs non-FDR

*p < 0.05, **p < 0.01, ***p<0.001

FDR, first-degree relatives of type 2 diabetes mellitus; HFD, high-fat diet.

Supplementary table S3. Metabolic profile changes (after - before high fat diet intervention) stratified by BMI

Variables	**BMI < 25**			**BMI >=25**		
	FDR (N=15)	Non-FDR (N=19)	P	FDR (N=15)	Non-FDR (N=11)	P
Fasting insulin changes (μIU/mL)	1.72±2.82	1.94±2.96	0.830	2.22±4.64	0.76±2.96	0.371
HOMA-IR changes	0.33±0.61	0.35±0.61	0.928	0.63±1.23	0.16±0.77	0.277
Serum uric acid changes (mg/dL)	0.04±0.76	0.08±0.67	0.875	0.48±0.87	-0.70±0.71	**0.001*** **

All values are expressed in mean±SD or median (IQR)

**p<0.01, ***p<0.001

BMI, body mass index; FDR, first-degree relatives of type 2 diabetes mellitus; HOMA-IR, homeostatic model assessment for insulin resistance

Supplementary table S4. Metabolic profile changes (after – before high fat diet intervention) stratified by central obesity

Variables	WC < 90			WC >= 90		
	FDR (N=16)	Non-FDR (N=24)	P	FDR (N=14)	Non-FDR (N=6)	P
Fasting insulin changes (μIU/mL)	1.69±3.77	1.30±2.96	0.721	2.29±3.92	2.32±3.10	0.990
HOMA-IR changes	0.37±0.94	0.29±0.68	0.859	0.61±1.01	0.24±0.67	0.433
Serum uric acid changes (mg/dL)	-0.03±0.75	-0.13±0.76	0.696	0.59±0.83	-0.55±0.82	**0.011***

All values are expressed in mean±SD or median (IQR)

*p<0.05

WC, waist circumference; FDR, first-degree relatives of type 2 diabetes mellitus; HOMA-IR, homeostatic model assessment for insulin resistance
